# A Novel Splicing Mutation of *KIT* Results in Piebaldism and Auburn Hair Color in a Chinese Family

**DOI:** 10.1155/2013/689756

**Published:** 2013-08-13

**Authors:** Yong-jia Yang, Rui Zhao, Xin-yu He, Li-ping Li, Ke-wei Wang, Liu Zhao, Ming Tu, Jin-song Tang, Zhi-guo Xie, Yi-min Zhu

**Affiliations:** ^1^The Laboratory of Genetics and Metabolism, Hunan Children's Research Institute (HCRI), Hunan Children's Hospital, The Paediatric Academy of University of South China, Changsha 410008, China; ^2^The Laboratory of Basic Medicine, Hunan Children's Research Institute (HCRI), Hunan Children's Hospital, The Paediatric Academy of University of South China, Changsha 410008, China; ^3^The Department of Research, Hunan Children's Research Institute (HCRI), Hunan Children's Hospital, The Paediatric Academy of University of South China, Changsha 410008, China; ^4^Institute of Mental Health, Second Xiangya Hospital, Central South University, Changsha 410008, China; ^5^Institute of Endocrinology Mental Health, Second Xiangya Hospital, Central South University, Changsha 410008, China; ^6^Department of Emergency, Hunan Children's Hospital, The Paediatric Academy of University of South China, Changsha 410008, China

## Abstract

Piebaldism is a rare autosomal dominant disorder of melanocyte development, which is mostly caused by *KIT* gene. The key characteristics of piebaldism include localized poliosis, congenital leukoderma, and other variable manifestations. The previous study has illustrated that the homogeneous *MC1R* (a gene which is associated with the hair color) variant (p.I120T) coordinating with *KIT* mutation may lead to auburn hair color and piebaldism. In this study, we have investigated a Chinese family with piebaldism and auburn hair color; the mutation screening of *KIT* and *MC1R* genes identified that only a splicing mutation (c. 2484+1G>A) of *KIT* gene cosegregated with the auburn hair color and piebaldism. The data of this study and others suggests that the KIT mutation may causes of the auburn hair color in the piebaldism patients.

## 1. Introduction

Piebaldism (OMIM 172800) is rare autosomal dominant disorder of melanocyte development, which is mostly caused by *KIT* gene (stem cell growth factor receptor gene, also known as a protooncogene, NM_000222.2) mutations [1]. 

The key characteristics of piebaldism include localized poliosis and congenital leukoderma (principally affecting the forehead, ventral trunk, and limb extremities), and the patches are usually stable throughout life [1]. 

Other variable manifestations of *KIT*-mutated piebaldism include the following: (1) café spots or hyperpigmented spots exist in the depigmented skin area and can develop at the margins or within the macules [1]; (2) in mild form of piebaldism, the leukoderma was very small, a white forelock cannot be seen, and in certain patients even the main feature of leukoderma can be an incomplete penetration [2]; (3) *KIT* mutation correlates with other modifier genes in a specific patient, leading to the hair colour change [3]. 

Recently, Oiso et al. described a 1-year-old Japanese girl who presented with piebaldism and auburn hair color [3]. The mutation screening of *KIT* and *MC1R* genes (*MC1R* was a previously identified gene which was associated with red hair in recessive mode, NM_002386.3 [4]) disclosed a novel mutation p.P832L in *KIT* and coexistence of a homozygous variant p.I120T in *MC1R* gene [3]. The p.I120T variant was previously deemed as a polymorphism, and the incidence of the homozygous p.I120T of *MC1R* was one person per 2268 Japanese people [5]. It is then raising the possibility that the homozygous p.I120T of *MC1R* (modifier gene [6]) correlating with KIT p.P832L mutation results in new phenotype of auburn hair colour.

In this study, we have investigated a Chinese family with piebaldism and auburn hair colour; the mutation screening of *KIT* and *MC1R* genes has identified only a splicing mutation (c. 2484+1G>A) in *KIT* gene that cosegregated with the piebaldism and auburn hair color in the family.

## 2. Materials and Methods 

**Figure 1:**
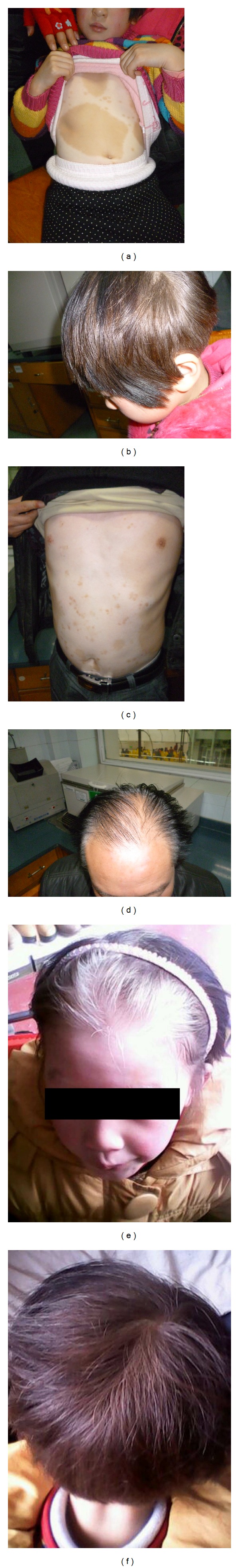
The clinical manifestation of the Chinese family with piebaldism and auburn hair color. ((a), (b)) The individual III:2. ((c), (d)) The individual II:3. ((e), (f)) The individual III:1.

A Chinese Han family with five members affected by piebaldism and auburn hair color (Figure 1(a)) and 60 healthy controls (31 males and 29 females) were included in this study. All adult individuals and the parents of the minors who participated in this study gave written informed consent, which was approved by the Ethics Committee of the Hunan Children's Hospital, Changsha China. The procedures of the committee conformed to the principles of the declaration of Helsinki, 2008 edition.

Genomic DNA was extracted from peripheral blood (2 mL in heparin sodium tubes) using the phenol/trichloromethane method prescribed by standard protocol. All specimens were quantified by spectrophotometry and diluted to 50 ng/*μ*L for polymerase chain reaction (PCR). 

The coding regions and the intron/exon junctions of the *KIT* and *MC1R* genes were amplified by PCR using the primers synthesized by local biotech company and designed using the software Primer3 (http://frodo.wi.mit.edu; primers and PCR conditions available on request). 

Sequencing reaction (BigDye 3.1 Kit, Applied Biosystems, USA) of the purified PCR products was carried out according to the recommended procedures. The labeled PCR fragments were purified through 70% alcohol precipitation and electrophoresed on an ABI-A3500 genetic analyzer (Applied Biosystems, USA).

All the results were compared with the reference (*KIT*: NM_000222.2, *MC1R*: NM_002386.3, http://genome.ucsc.edu/cgi-bin/hgGateway) using SEQMAN software (DNA Star Package, WI, USA). 

For splicing analysis, total RNA was isolated from peripheral blood of a patient (III:2) and a control (II:4) using RNApure Blood Kit (CWBIOTECH, Beijing, China). First strand cDNA was produced by HiFi-MMLV cDNA Kit (CWBIOTECH, Beijing, China) according to the manufacturer's recommendations. 

RT-PCR primers (F: 5′-TGACGAGTTGGCCCTAGACT-3′; R: 5′-GAAGCCTTCCTTGATCATCTTG-3′; the predicted product size was 386 bp) were designed according to the cDNA sequence of *KIT* gene (NM_000222.2) which flanked exons 17 and 18 of *KIT*. The PCR products were electrophoresed on a 6% polyacrylamide gel. The dissected bands were purified by standard methods and sequenced on a 3500 genetic analyzer which is mentioned previously.

## 3. Results

**Figure 2:**
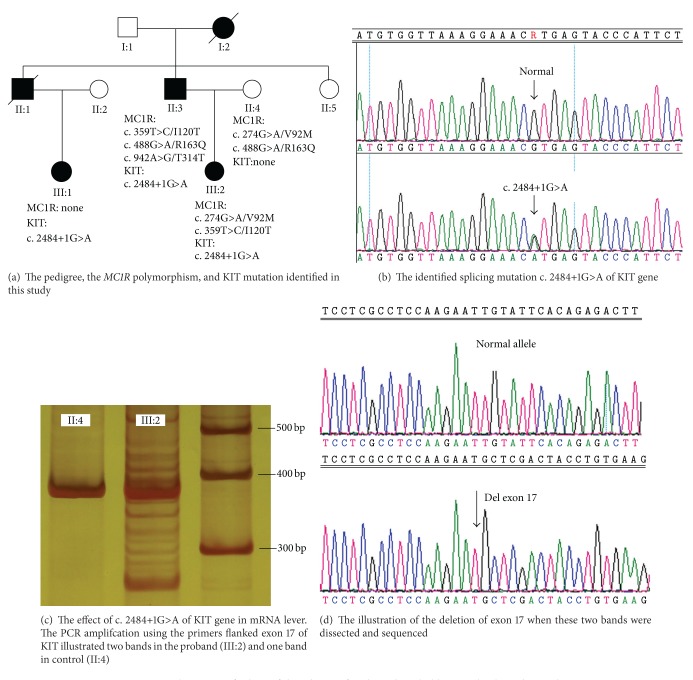
The genetic finding of the Chinese family with piebaldism and auburn hair color.

The proband (Figure 2(a), III:2) was a 10-year-old girl, who came to our laboratory for chromosome analysis (due to her multiple malformations, as her parents described). Physical examination revealed that (1) there is a prominent leucoderma on the ventral trunk (Figure 1(a)), knees, elbows, and forehead; (2) multiple hyperpigmented spots exist in the patches of her skin (Figure 1(a)); (3) the most of her hair was auburn color, which was mixed with a few of white hair and a very few of black hair (Figure 1(b)); and (4) a prominent short stature exists (117.7 cm: normal 137.2 cm). Her result for chromosome G band analysis was 46, XX. Her father (Figure 2(a), II:3, at the age of 35 years old) also had leucoderma on the ventral anterior trunk, knees, elbows, and a prominent poliosis of hair, eyebrows, and eyelashes (Figures 1(c) and 1(d)). As he described, a small but obvious patch of his posterior hair was also auburn color (unfortunately, due to his long-term hair dyeing, the picture of his auburn color hair was unavailable).

The III:1 (Figure 2(a)) was a 9-year-old girl who presented with leucoderma on the ventral trunk, knees, elbows, a prominent white forelock, and poliosis of eyebrows and eyelashes (Figure 1(e)). Except for her frontal forelock and a very small number of black hairs in the middle scalp, the remaining hair color was auburn (Figure 1(f)).

Her father (Figure 1(a), II:1, as she described, had deafness detailed clinic data was unavailable) and her grandmother (Figure 2(a), I:2) also had typical piebaldism phenotype, but the detail of hair color was unavailable. 

A heterozygous splicing mutation in intron 17 (c. 2484+1G>A or IVS17+1G>A, Figures 2(a) and 2(b)) was firstly detected in the three affected family members, and it was not detected in II:4 (Figure 2(a)) and 60 ethnically matched control samples by direct sequencing.

The mutation screening of *MC1R* gene in all available family individuals (II:3, II:4, III:1, and III:2, Figure 1(a)) detected 4 heterozygous variants of *MC1R*, including c.274G>A, c.359T>C, c.488 G>A, and c.942 A>G and identified neither heterogeneous nor homogeneous variant co-segregated with auburn color hair in the family (for detailed variants distribution see Figure 2(a)). 

The PCR products of RT-PCR were electrophoresed on a 6% polyacrylamide gel (Figure 2(c)). In patients (III:2, Figure 2(a)) two bands were visualized (Figure 2(c)) while in control (Figure 2(a)) only one band detected. 

These prompted us was dissect to these two bands and sequence them on a 3500 genetic analyzer, respectively. The results revealed a heterogenous deletion of 123 base pairs (Figure 2(d), which coincides with all sequences of exon 17 of *KIT*). This result indicated that the splicing mutation (c. 2484+1G>A) skipped exon 17 of *KIT* gene (Figure 2(d)) which leads to a 41 amino acids deletion from the amino acids 788 to 828 of KIT protein in the family.

## 4. Discussion

The KIT receptor contains seven domains, including a signal sequence (SS, amino acids 1–22), an amino-terminal extracellular ligand-binding domain (EC, amino acids 23–520), a transmembrane domain (TM, amino acids 521–543), a juxtamembrane domain (JM, amino acids 544–581), and two TK domains (TK1, amino acids 582–684 and TK2, amino acids 762–973) separated by a kinase insert domain (KI, amino acids 685–761) [7, 8]. The last four domains also were known as the cytoplasmic domains or intracellular domains [7]. Within TK2 domain, the amino acid residue 810–839 (an activation loop) was a highly conserved enzymatic site [6]. The binding of KIT ligand (KITLG) to the extracellular domain of KIT leads to the receptor dimerization, the intracellular autophosphorylation, and then tyrosine kinase activation (TKA) [9]. 

The TKA was the final executed step in the KIT banding process, which triggers the regulations of the migration of melanocytes, cell proliferation, differentiation, survival, melanogenesis, and melanosome transfer [10].

Patients with the mutation that occurred in extracellular domain of KIT usually manifested relatively mild form of piebaldism as those of heterogeneous mutations (missense or a mutation of complete elimination of the production of KIT by the defective allele) preserved 50% or more of the KIT function [7]. 

In contrast, the majority (87%) of the most severe forms of KIT-mutated piebaldism (which is well illustrated in Figure 1 of the paper by Murakami et al. [7]) were caused by mutations that occurred in intracellular domain, especially in TK domains, as this kind of mutations tends to be levying dominant negative effects and haploinsufficiency effects, which disrupts the TKA process and leads to only 25% or less of KIT function [7].

The splicing mutation (c. 2484+1G>A) identified in this study was at the donor splice sites of exon 17 of KIT, which leads to the deletion of amino acids 788 to 828 of KIT protein (which was deduced by the mRNA analysis of the propositus in here). This deletion was 19 amino acids that overlapped with the highly conserved enzymatic site (amino acids: 810–839 activation loop) of the TK2 domain of KIT. 

Recently, Oiso et al. described a 1-year-old Japanese girl who presented with the severe form of piebaldism and auburn hair color [3]. The authors proposed that the severe piebaldism was caused by the missense mutation (p.P832L) of *KIT*, and the auburn hair color phenotype was caused by the coordinating effects of the *KIT* mutation and the homozygous variant p.I120T of MC1R. Of note, the *KIT* p.P832L mutation was also located in the area of the highly conserved enzymatic site (amino acids: 810–839 activation loop) of the TK2 domain of KIT.

The *MC1R* gene encoded a melanocortin-1 receptor, which was located on the plasma membrane of melanocytes, and participated in the production of the pigment melanin through a process referred to as melanogenesis. The MC1R protein is a seven transmembrane G-protein coupled receptor and identified as one of the key proteins involved in regulating mammalian skin and hair color [11].

In human population, the *MC1R* gene (MIM155555) has many polymorphisms; some of them have been identified with association with red hair, fair skin, freckling, and increased skin cancer risk [12, 13].

The previous study has clearly shown that the majority of persons with red hair are either homozygous or compound heterozygous for a combination of the *MC1R* variants, such as the variants R151C, R160W, and D294H [14]. Also, other 10–20% of individuals who had red hair (having lighter red-colored hair than those harboring two diminished function alleles) showed only a heterogeneous change of *MC1R* [15].

In this study, we have performed the sequencing analysis of *MC1R* gene in a Chinese Han family and identified successfully four polymorphisms (Figure 2(a)), which were previously reported in general population [5, 12]. In the family, the auburn hair color seems to be cosegregated with the piebaldism and the splicing mutation (c. 2484+1G>A) of *KIT* but not with any of the 4 polymorphisms of the *MC1R* gene. To our knowledge, a previous study of *KIT* mutation screening in Chinese population recently has also disclosed that a *KIT* mutation (Ala621Asp) leads to severe form of piebaldism as well as auburn hair colour in a female child [16]. It is unfortunately that the *MC1R* mutation screening was not performed in that patient [16].

It is interesting that the deafness was occurred in a family member (the individual II:1 (Figure 2(a)) who most probably carried the *KIT* mutation (c. 2484+1G>A) ) in this study. However, the previously study by Spritz and Beighton [17] had illustrated a heterogeneous *KIT* mutation (R796G) in a sporadically case associated with piebaldism and deafness. It is then probably that such kind of deafness with low-penetrance in the *KIT* heterogeneous-mutated patients may attributes to the different gene background of each individual. Further study for delineation of the deafness and *KIT* mutation is needed in the future. 

In this study, we have successfully identified a splicing mutation, which results in the deletion of exon 17 of *KIT*, and cosegregated it with two phenotypes of the severe form of piebaldism and the auburn hair color in a Chinese Han family. This data would expand the knowledge of the *KIT*-related phenotype-genotype correlations.
